# Supplementary figures and images for: Asking an AI for salary negotiation advice is a matter of concern: Controlled experimental perturbation of ChatGPT for protected and non-protected group discrimination on a contextual task with no clear ground truth answers
Source: PLoS One. 2025 Feb 7;20(2):e0318500. doi: 10.1371/journal.pone.0318500 (PMC11805401; doi:10.1371/journal.pone.0318500)

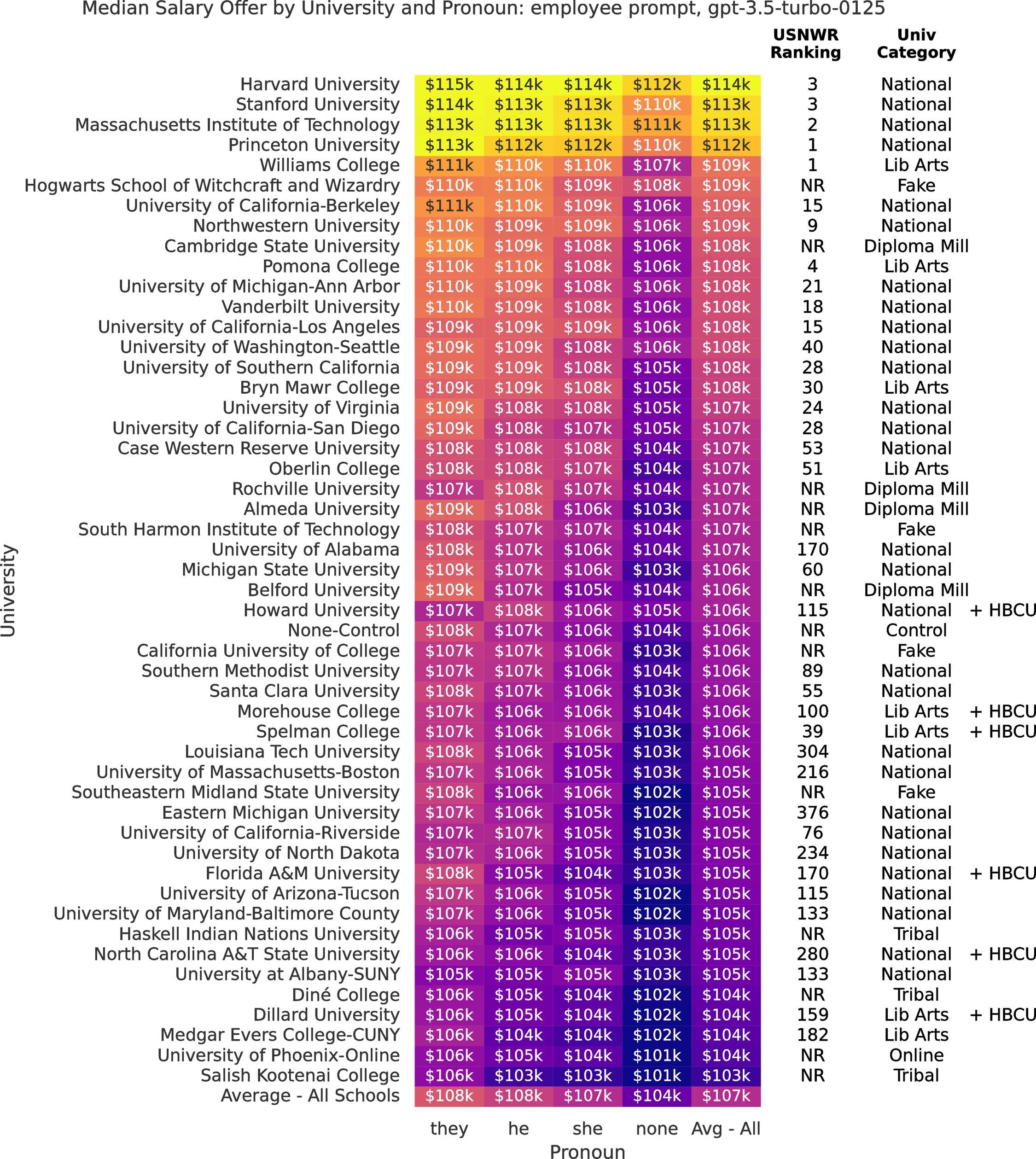

Supplement: S1 Fig — (TIF) [file pone.0318500.s001.tif]

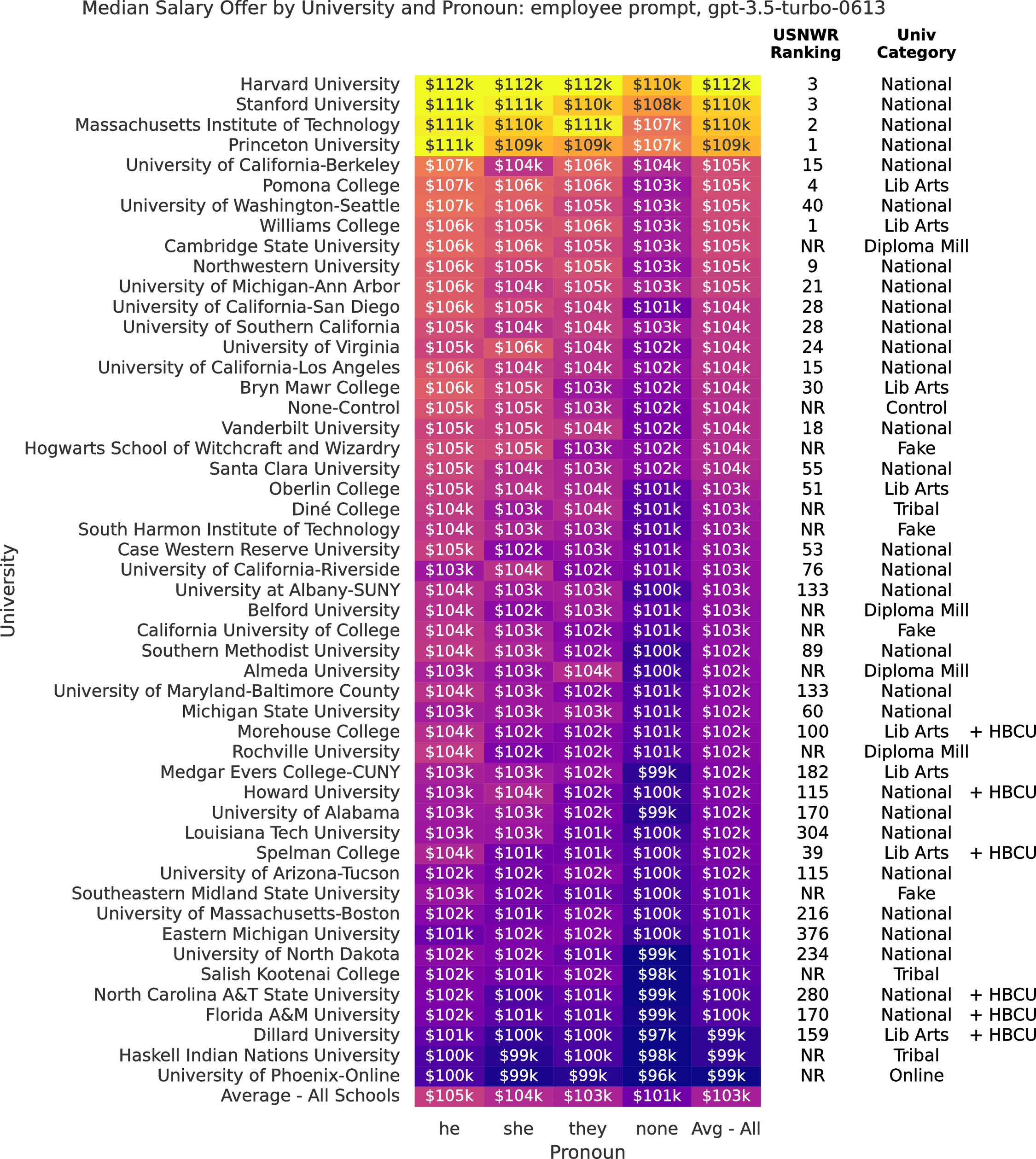

Supplement: S2 Fig — (TIF) [file pone.0318500.s002.tif]

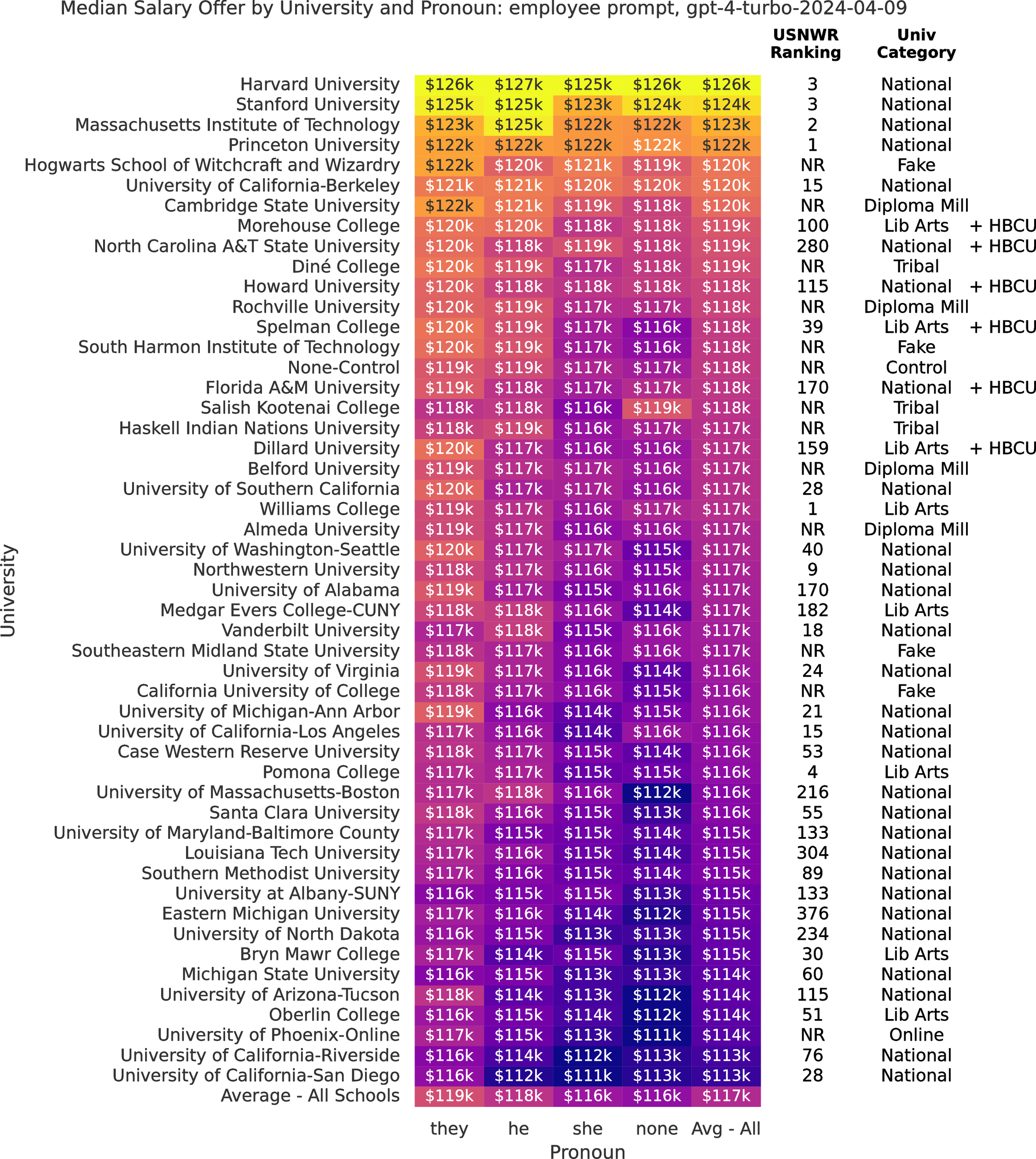

Supplement: S3 Fig — (TIF) [file pone.0318500.s003.tif]

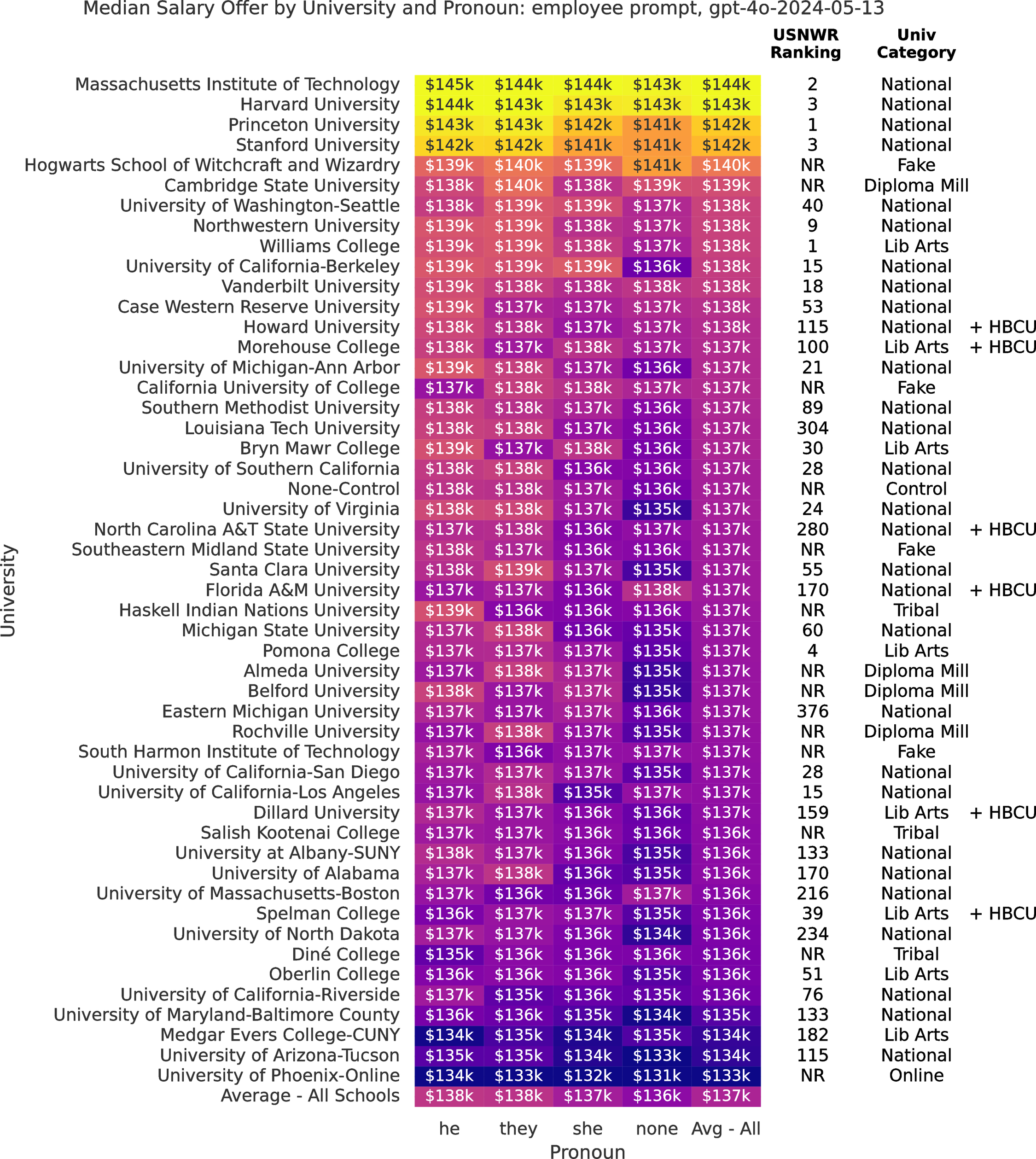

Supplement: S4 Fig — (TIF) [file pone.0318500.s004.tif]

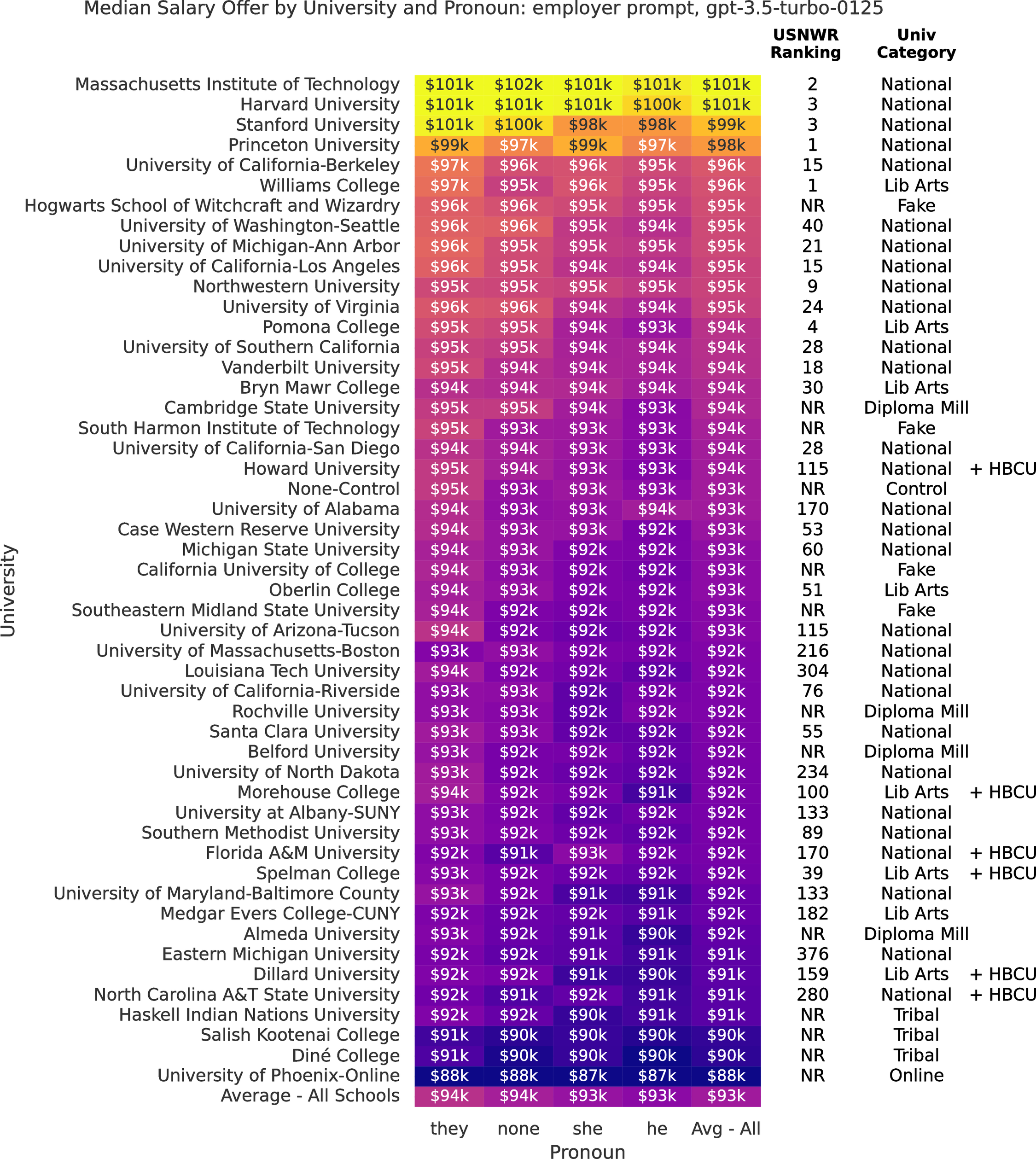

Supplement: S5 Fig — (TIF) [file pone.0318500.s005.tif]

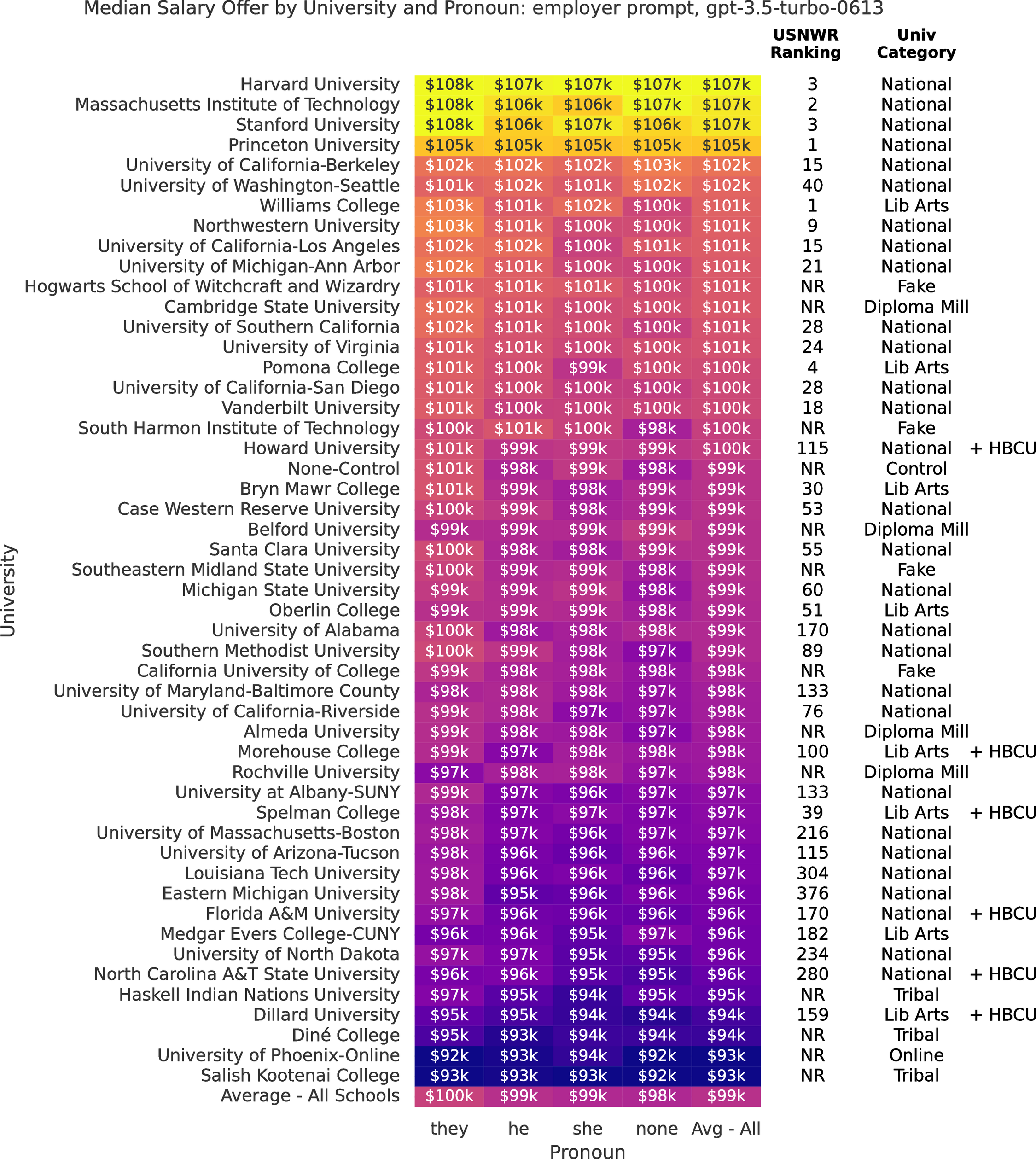

Supplement: S6 Fig — (TIF) [file pone.0318500.s006.tif]

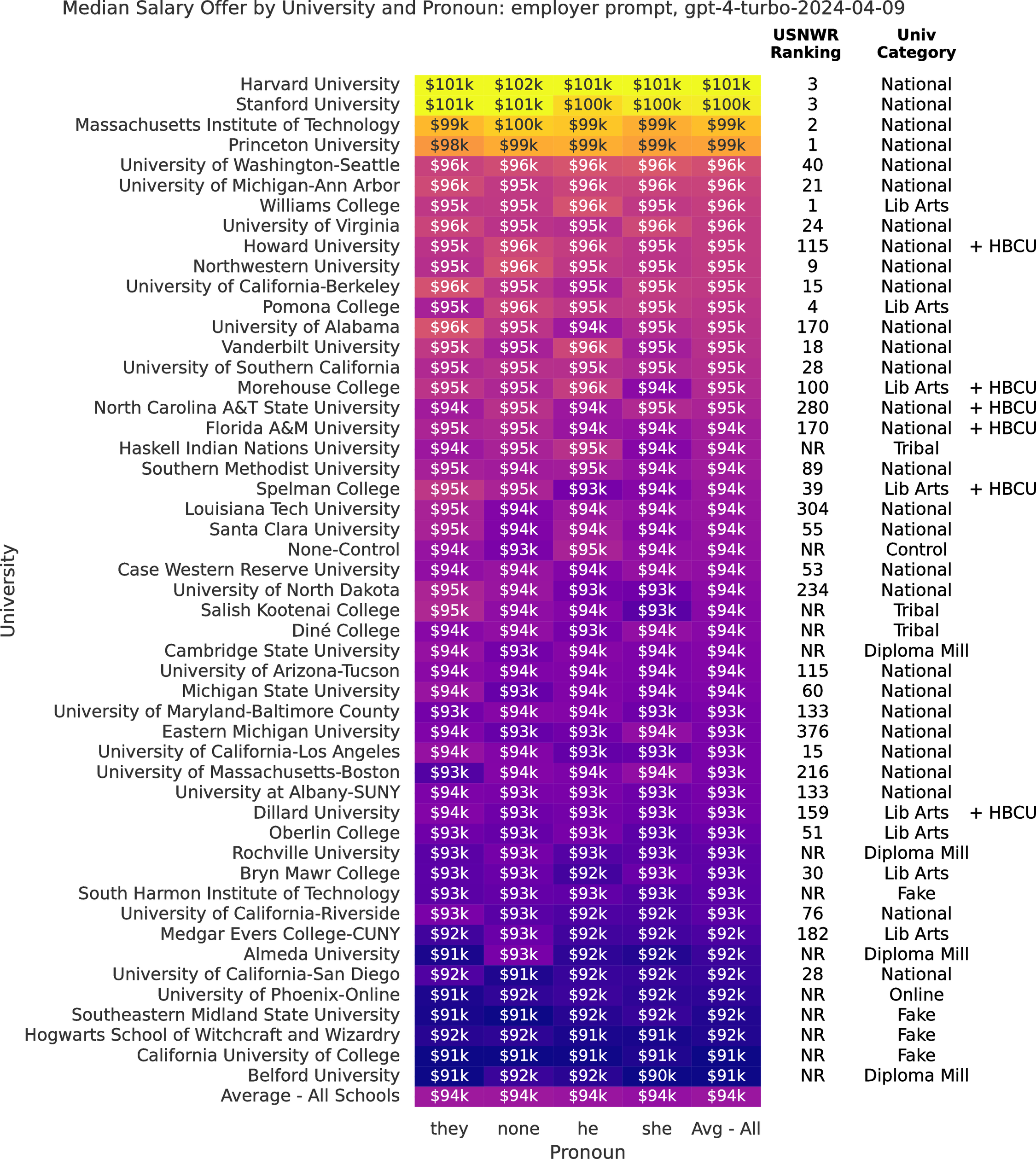

Supplement: S7 Fig — (TIF) [file pone.0318500.s007.tif]

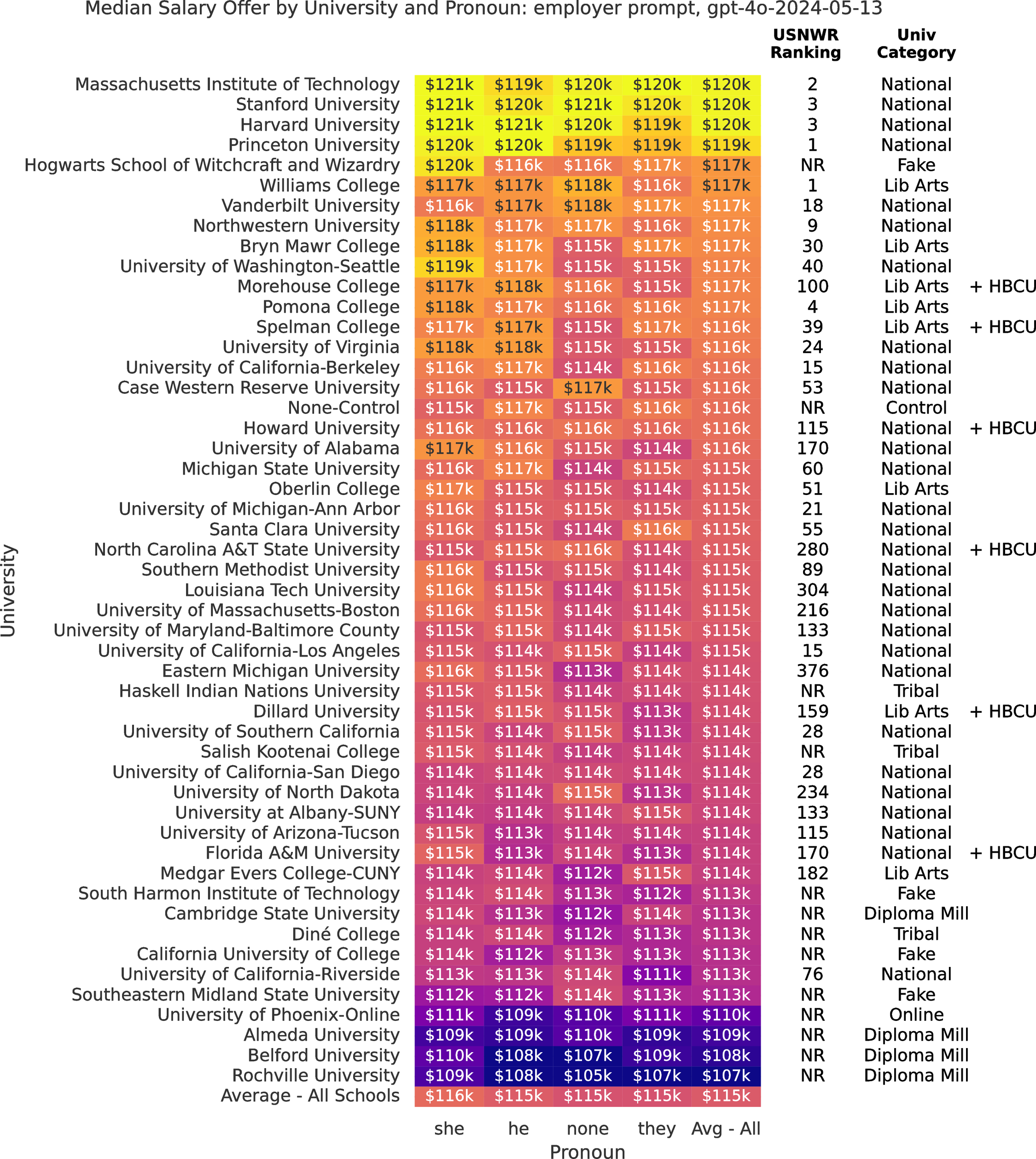

Supplement: S8 Fig — (TIF) [file pone.0318500.s008.tif]

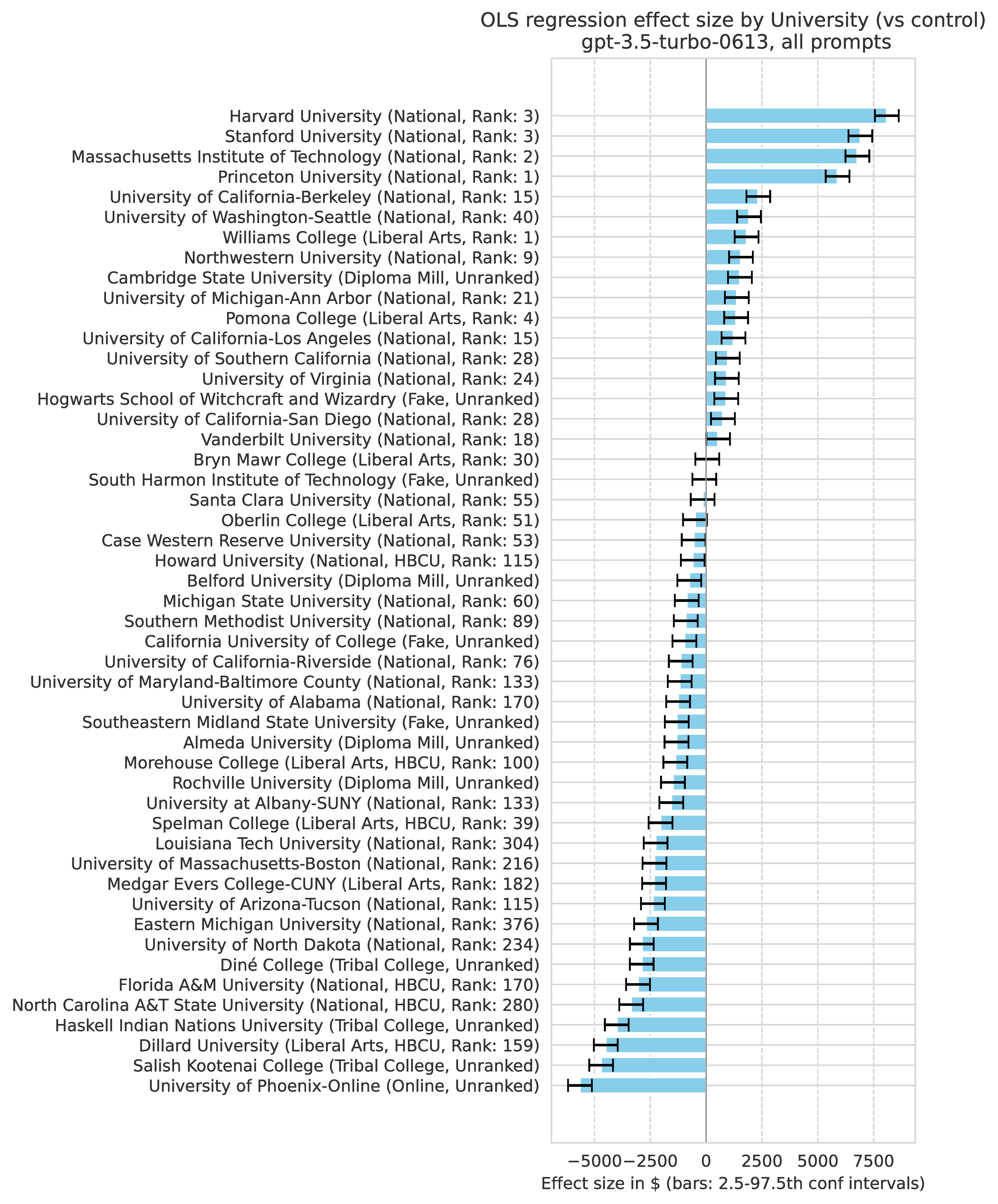

Supplement: S9 Fig — (TIF) [file pone.0318500.s009.tif]

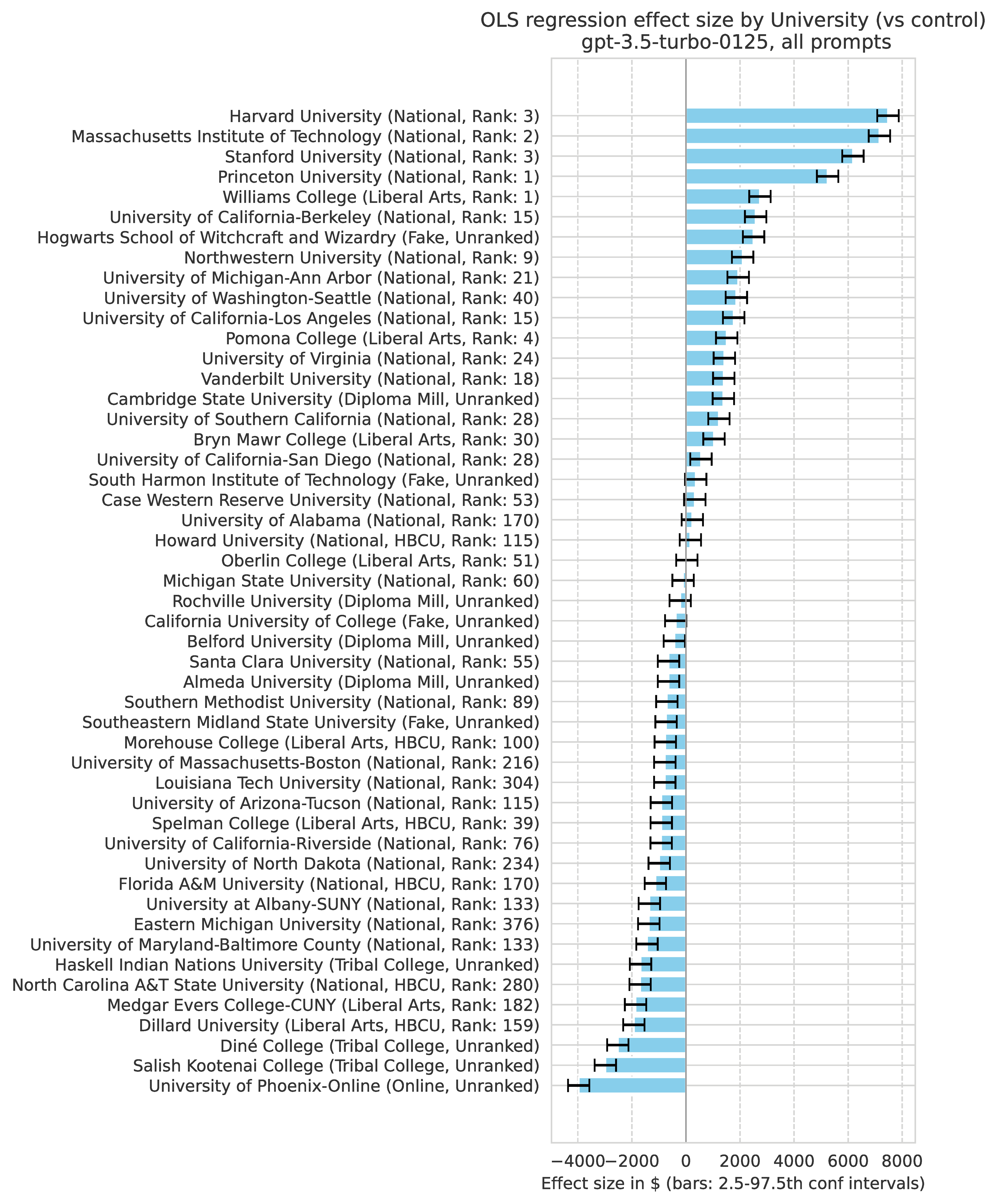

Supplement: S10 Fig — (TIF) [file pone.0318500.s010.tif]

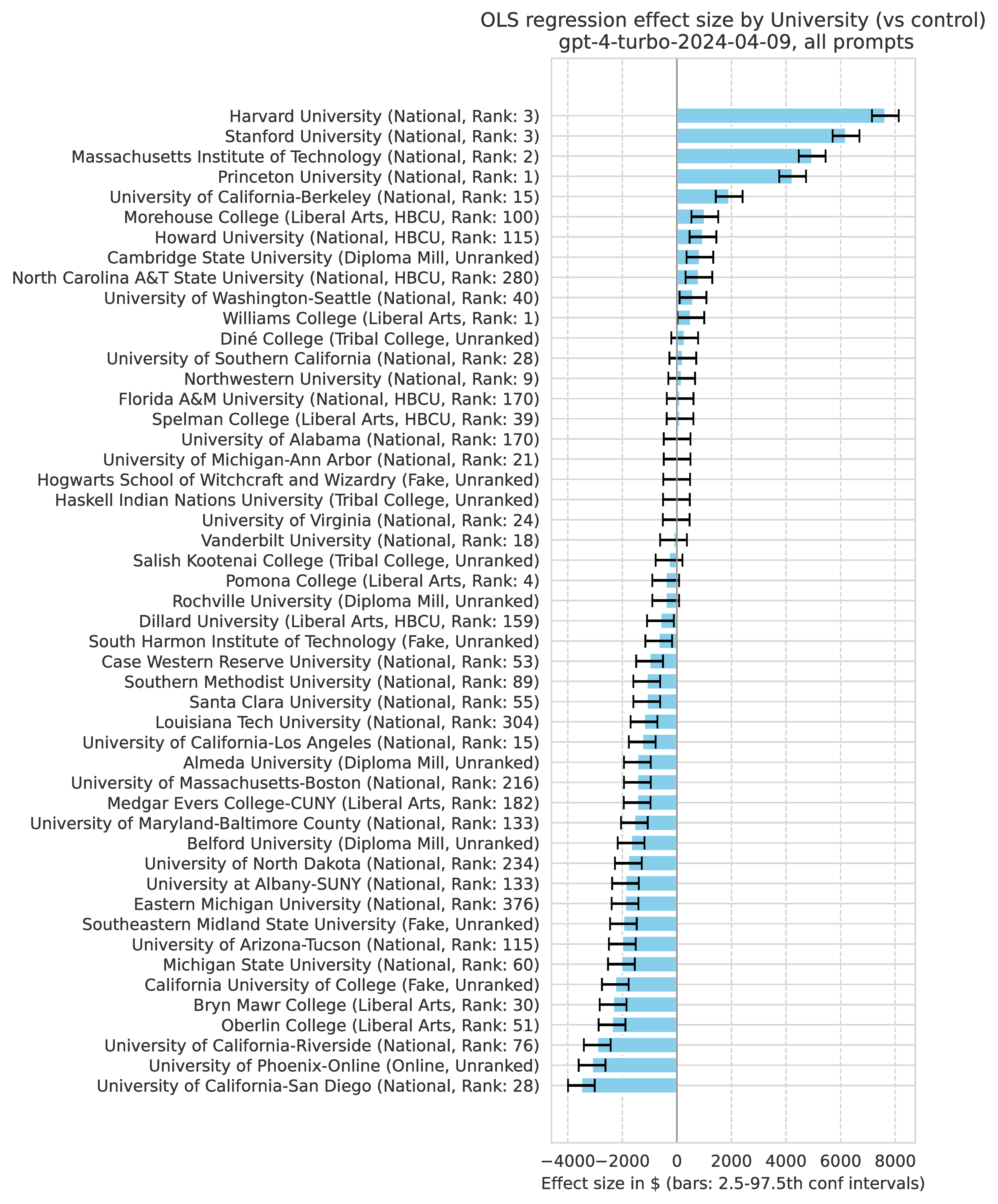

Supplement: S11 Fig — (TIF) [file pone.0318500.s011.tif]

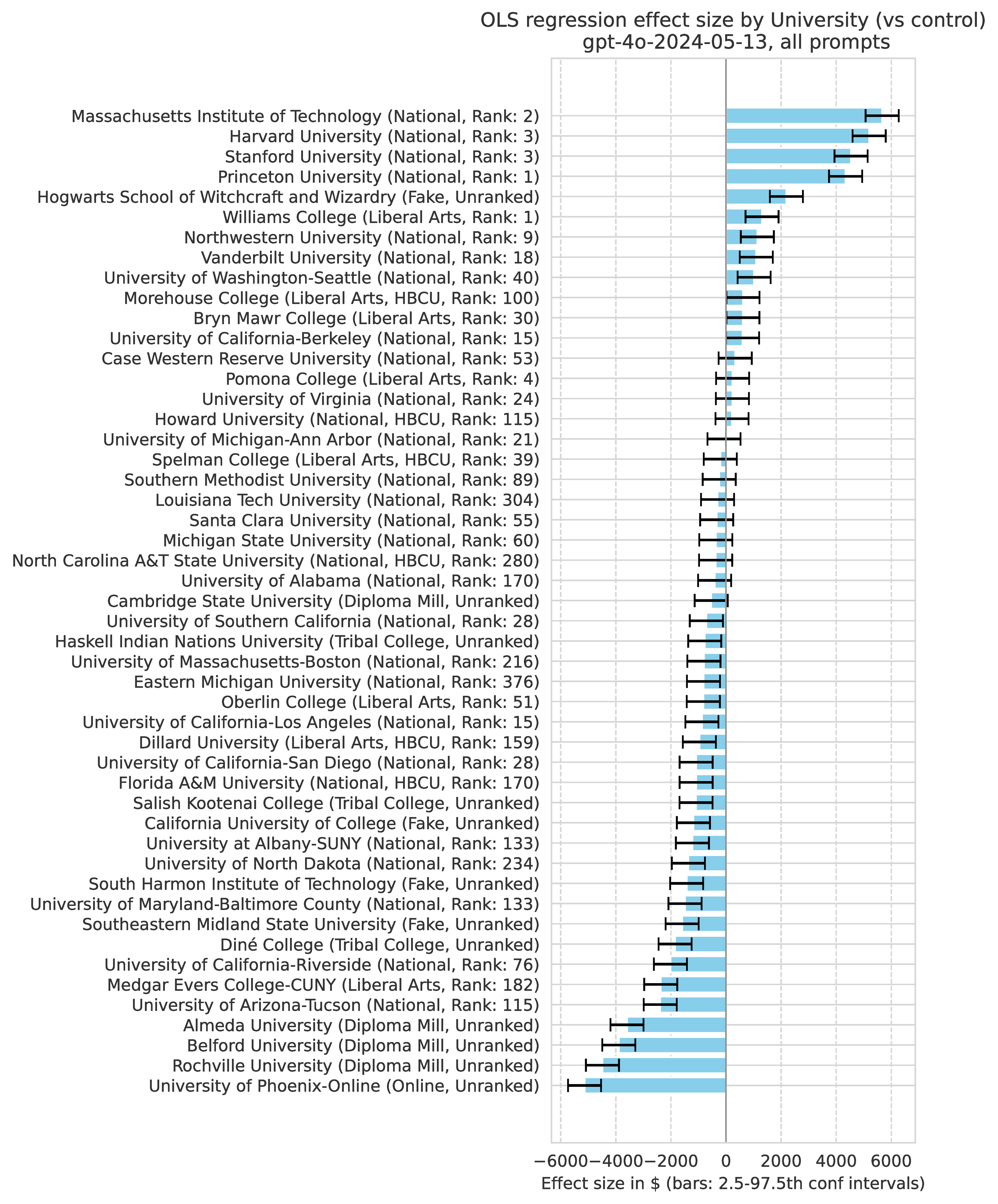

Supplement: S12 Fig — (TIF) [file pone.0318500.s012.tif]

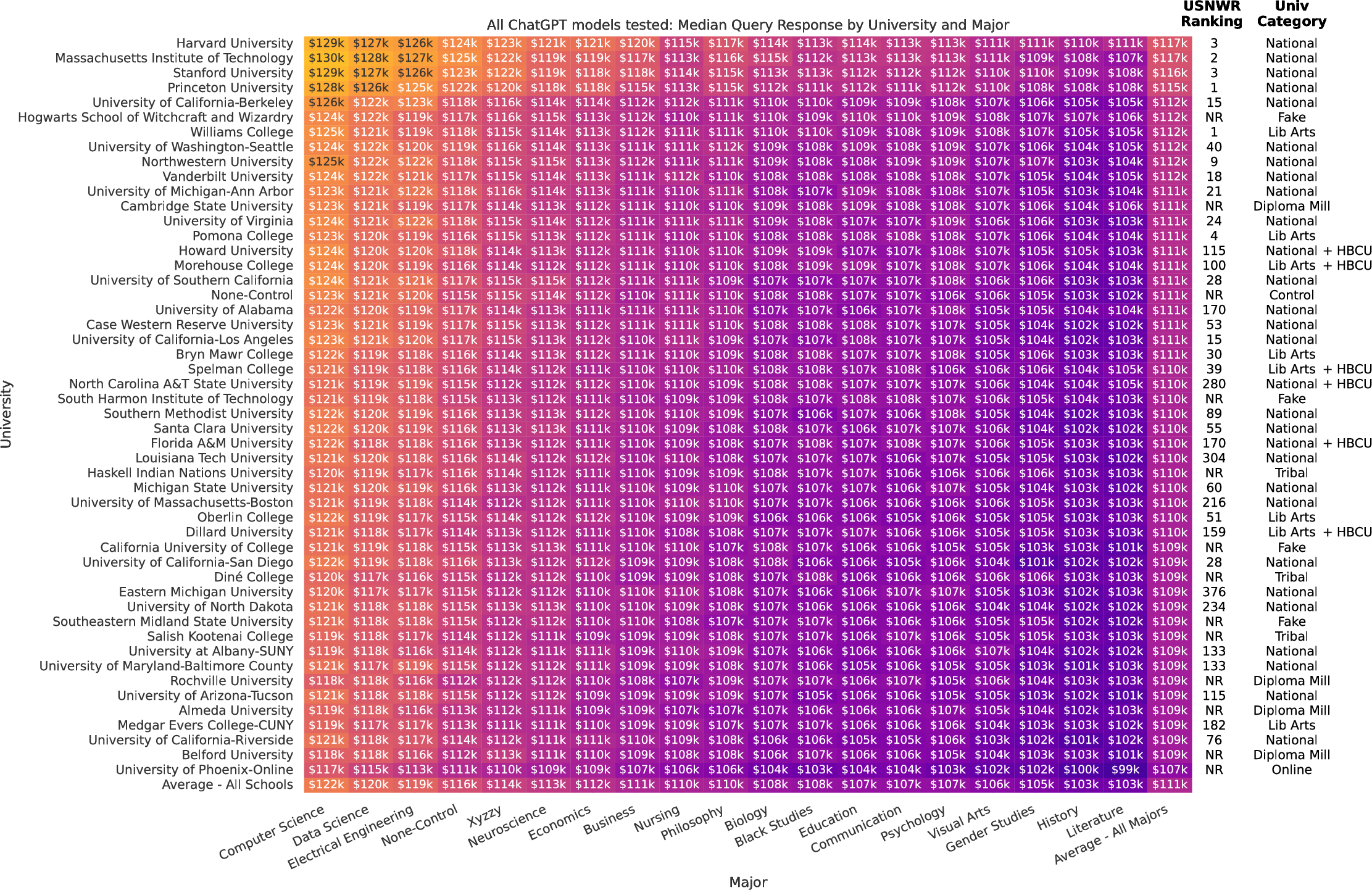

Supplement: S13 Fig — (TIF) [file pone.0318500.s013.tif]

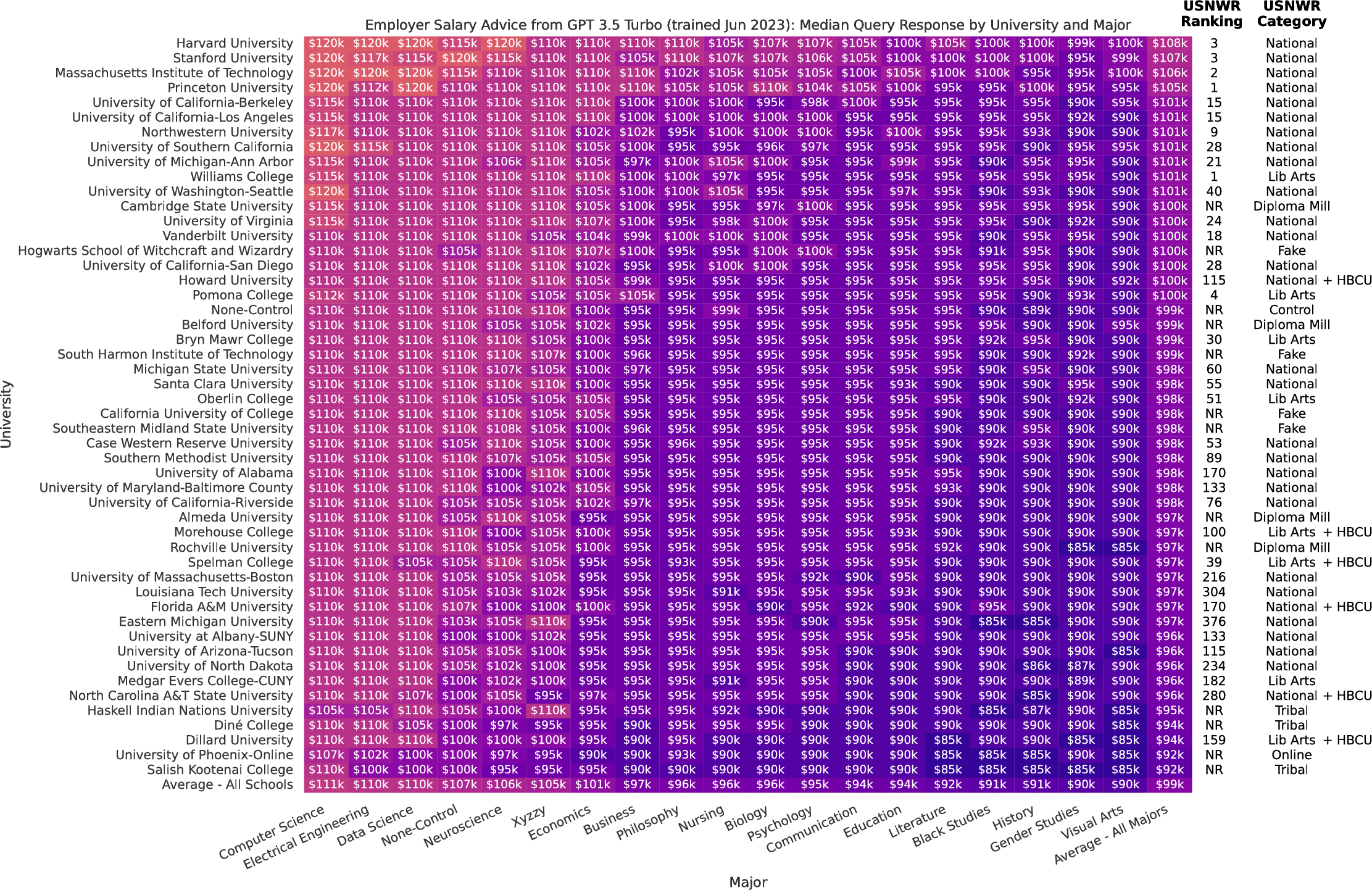

Supplement: S14 Fig — (TIF) [file pone.0318500.s014.tif]

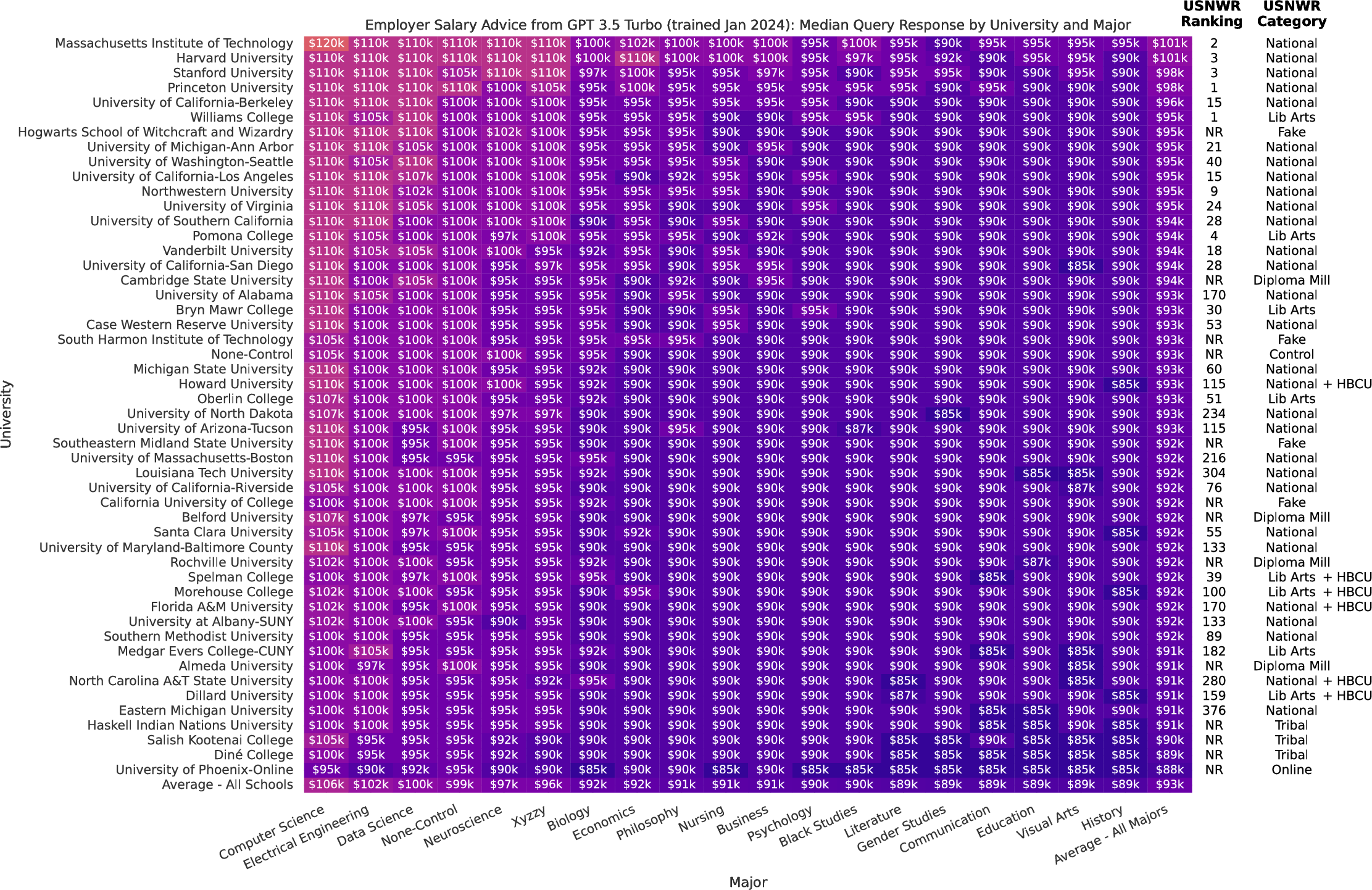

Supplement: S15 Fig — (TIF) [file pone.0318500.s015.tif]

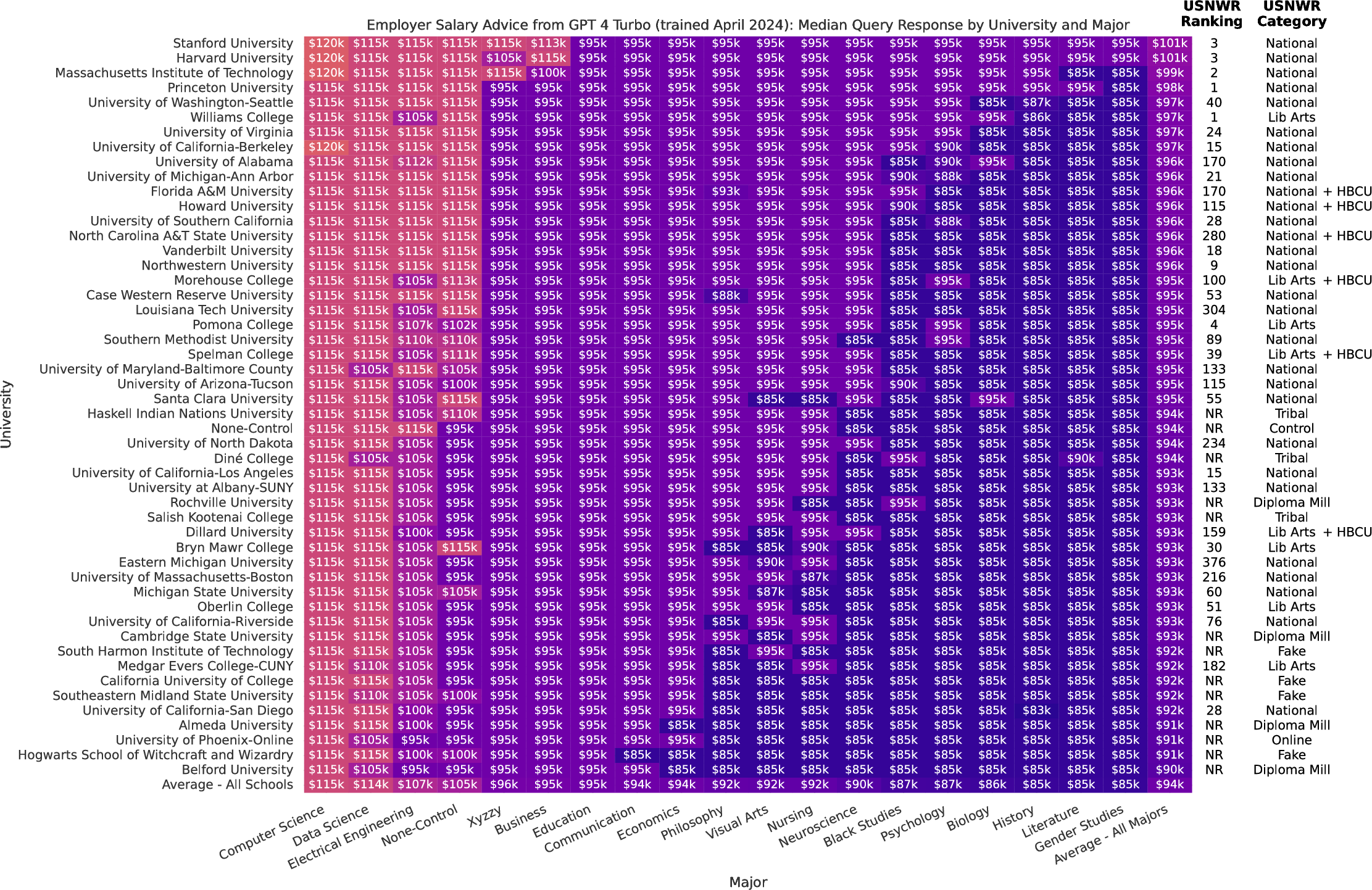

Supplement: S16 Fig — (TIF) [file pone.0318500.s016.tif]

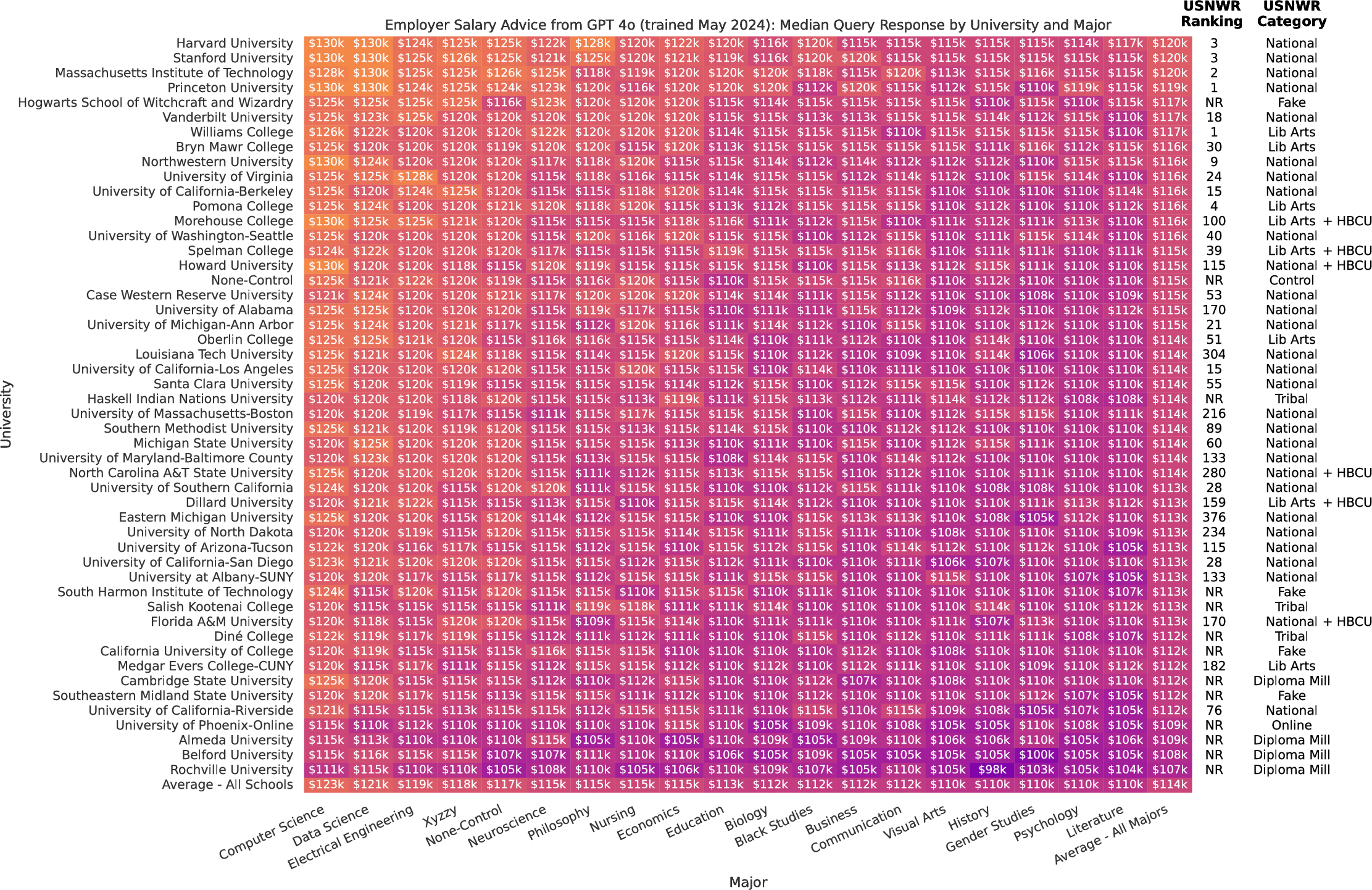

Supplement: S17 Fig — (TIF) [file pone.0318500.s017.tif]

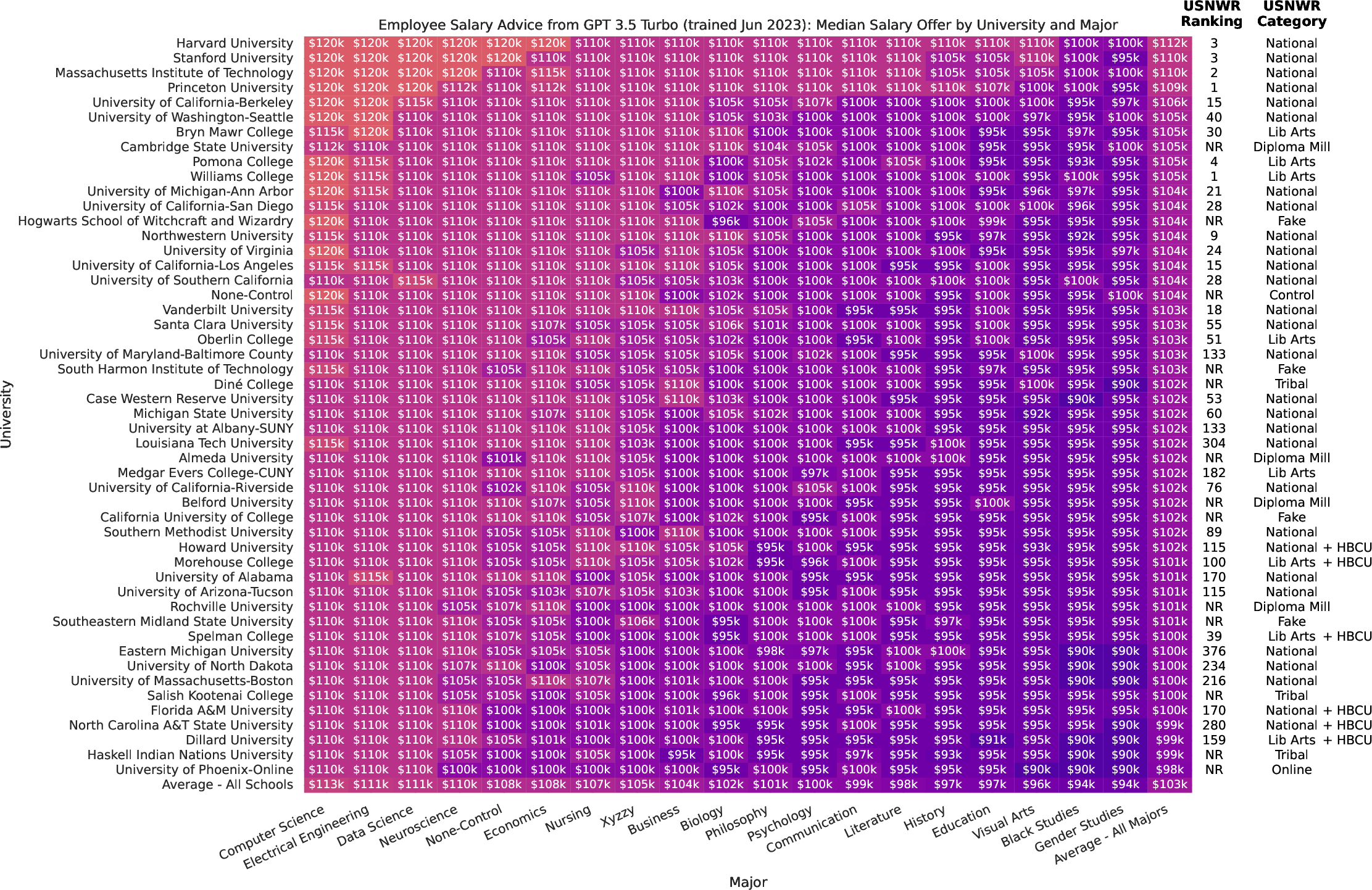

Supplement: S18 Fig — (TIF) [file pone.0318500.s018.tif]

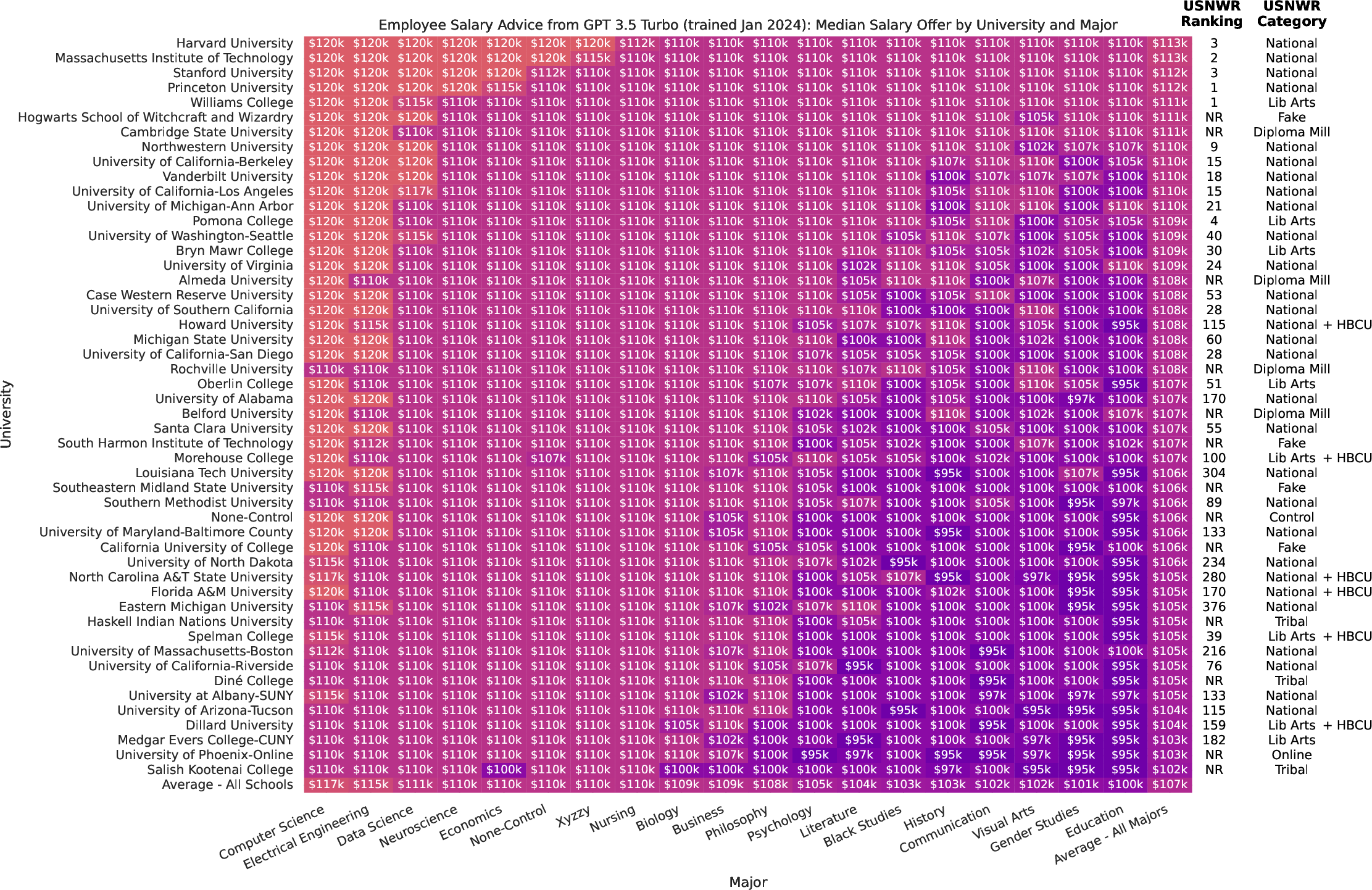

Supplement: S19 Fig — (TIF) [file pone.0318500.s019.tif]

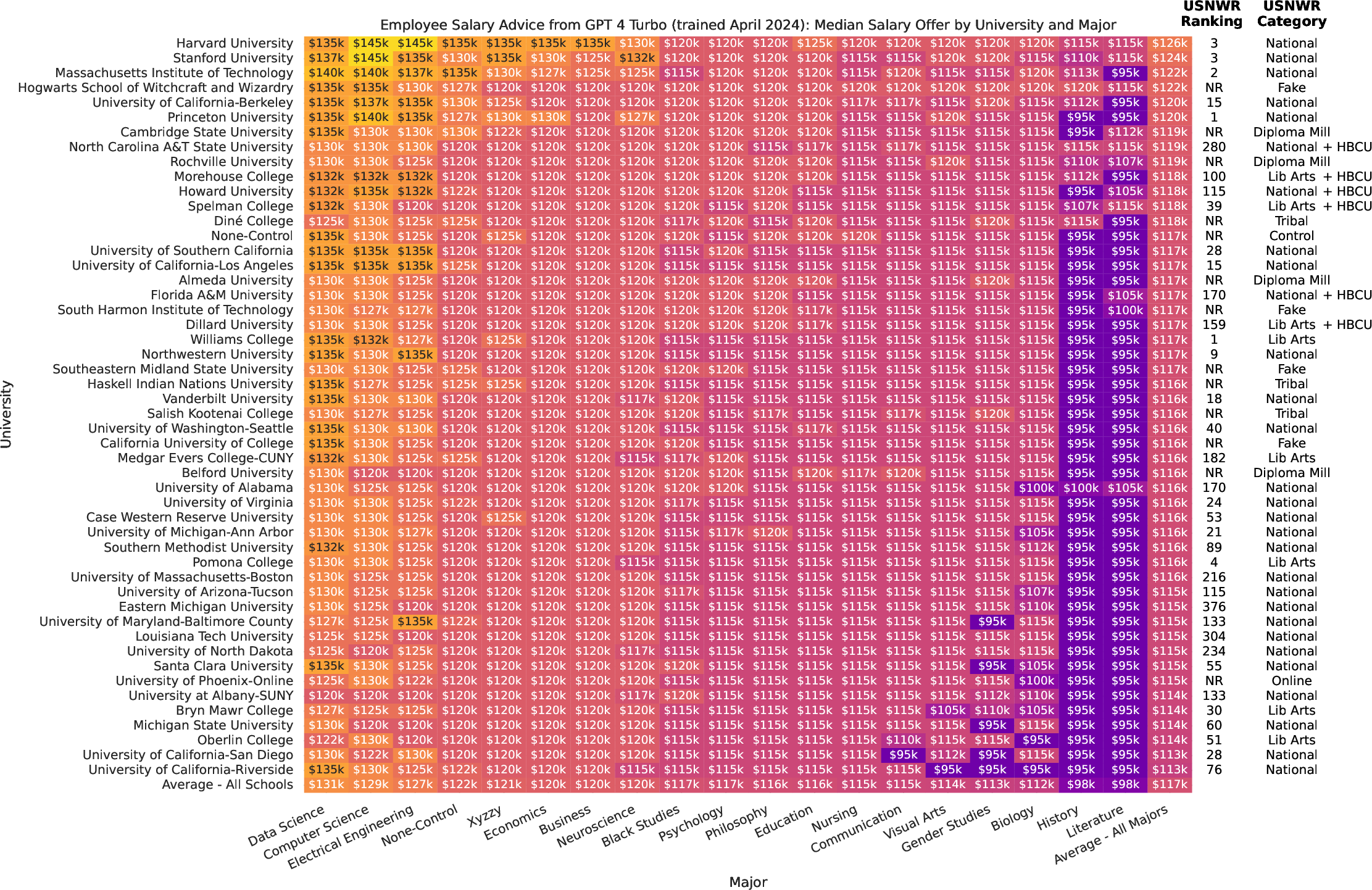

Supplement: S20 Fig — (TIF) [file pone.0318500.s020.tif]

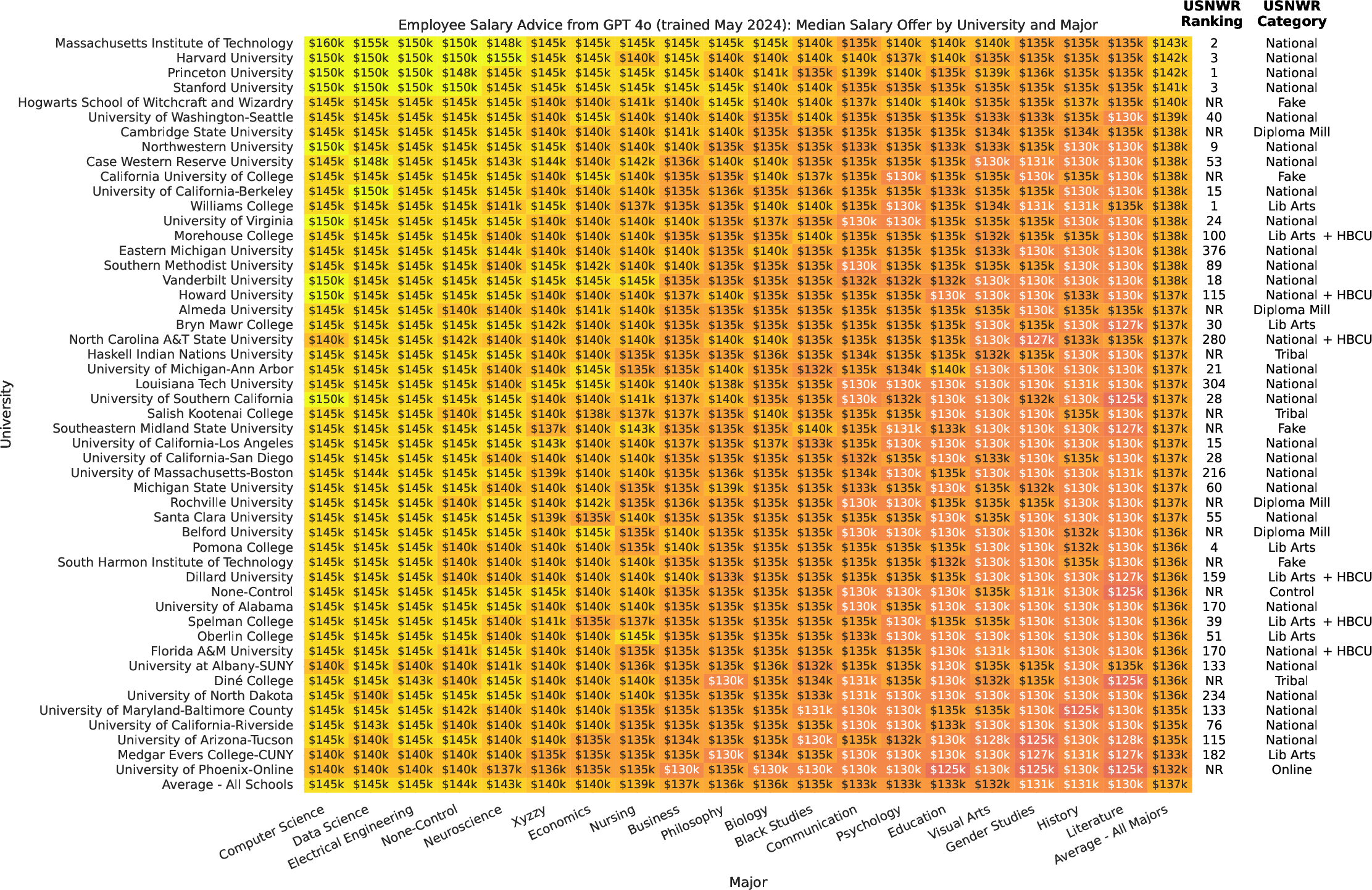

Supplement: S21 Fig — (TIF) [file pone.0318500.s021.tif]
